# Direct Detection by the Xpert MTB/RIF Assay and Characterization of Multi and Poly Drug-Resistant Tuberculosis in Guinea-Bissau, West Africa

**DOI:** 10.1371/journal.pone.0127536

**Published:** 2015-05-27

**Authors:** Paulo Rabna, Jorge Ramos, Gema Ponce, Lilica Sanca, Morto Mané, Ana Armada, Diana Machado, Fina Vieira, Victor F. Gomes, Elisabete Martins, Raffaella Colombatti, Fabio Riccardi, João Perdigão, Joana Sotero, Isabel Portugal, Isabel Couto, Jorge Atouguia, Amabélia Rodrigues, Miguel Viveiros

**Affiliations:** 1 Instituto Nacional de Saúde Pública/Projecto de Saúde de Bandim (INASA/PSB), Bissau, Guiné-Bissau; 2 Grupo de Micobactérias, Unidade de Microbiologia Médica, Global Health and Tropical Medicine (GHTM), Instituto de Higiene e Medicina Tropical, Universidade NOVA de Lisboa (IHMT, UNL), Lisboa, Portugal; 3 Unidade de Clínica Tropical, Instituto de Higiene e Medicina Tropical de Lisboa/Universidade NOVA de Lisboa (IHMT/UNL), Lisboa, Portugal; 4 Centro de Malária e Outras Doenças Tropicais (CMDT), Instituto de Higiene e Medicina Tropical de Lisboa/Universidade NOVA de Lisboa (IHMT/UNL), Lisboa, Portugal; 5 Hospital Raoul Follereau, Bissau, Guiné-Bissau; 6 Ministério da Saúde/Programa Nacional de Luta contra a Tuberculose, Bissau, Guiné-Bissau; 7 Aid, Health and Development-Onlus—Ahead-Onlus, Rome, Italy; 8 Centro de Patogénese Molecular, URIA, Faculdade de Farmácia, Universidade de Lisboa, Lisboa, Portugal; Barcelona University Hospital, SPAIN

## Abstract

**Background:**

This study aimed to evaluate the usefulness of the Xpert MTB/RIF assay for the rapid direct detection of *M*. *tuberculosis* complex (MTBC) strains and rifampicin resistance associated mutations in a resource-limited setting such as Guinea-Bissau and its implications in the management of tuberculosis (TB) and drug resistant tuberculosis, complementing the scarce information on resistance and genotypic diversity of MTBC strains in this West African country.

**Methods and Results:**

This cross-sectional prospective study included 100 consecutive TB patients with positive acid-fast smears at two months of anti-tuberculosis treatment or in a re-treatment situation, between May and December 2012. Resistance to rifampicin was detected using the GeneXpert system and the Xpert MTB/RIF assay. MTBC isolates obtained with the BACTEC MGIT 960 system were tested for susceptibility to first- and second-line anti-tuberculosis drugs. Overall, the prevalence of multidrug-resistant tuberculosis (MDR-TB) was found to be 9 cases. Of these, 67% (6 patients) of confirmed MDR-TB cases had no past history of TB treatment and 33% (3 patients) were previously treated cases. Extensively drug-resistant TB was not found. Molecular typing of the MDR-TB strains revealed recent transmission patterns of imported MDR strains.

**Conclusions:**

The Xpert MTB/RIF assay was reliable for the detection of rifampicin resistant MTBC strains directly from sputum samples of patients undergoing first-line treatment for two months, being more trustworthy than the simple presence of acid-fast bacilli in the smear. Its implementation is technically simple, does not require specialized laboratory infrastructures and is suitable for resource-limited settings when a regular source of electricity and maintenance is available as well as financial and operation sustainability is guaranteed by the health authorities. A high prevalence of MDR-TB among patients at risk of MDR-TB after two months of first-line treatment was found, in support of the WHO recommendations for its use in the management of this risk group.

## Introduction

Despite all the scientific and technological advances, tuberculosis (TB) remains a serious and important global health problem [1]. The emergence of resistance to drugs used for the treatment of TB, and particularly MDR-TB, has become an obstacle to effective global control of tuberculosis [2]. Multidrug resistant TB, and in particular extensively drug resistant TB (XDR-TB), are more expensive to treat, with a survival rate in patients co-infected with HIV that is lower in comparison with drug susceptible TB. This poses a particular problem for countries with few resources where the prevalence of TB and HIV is high and the access to therapeutic regimens of first- and second-line is limited [3,4].

In Guinea-Bissau, a small West African country with a population of approximately 1.6 million people and one of the poorest countries in the world, TB is known as one of the leading causes of morbidity and mortality, with one of the highest incidence rates in the world (242/100,000 inhabitants) [5].

The increasing course of drug resistant TB, of paucibacillary and extra-pulmonary cases, as well as of HIV co-infection rates worldwide, demands for faster methods to detect TB and MDR-TB, a key factor in the management and treatment of TB patients.

Rapid detection of MDR-TB in clinical samples allows starting an early and proper treatment regimen and is essential to prevent transmission of MDR-TB. Thus, National Tuberculosis Control Programs seek to counteract this situation by implementing these rapid detection strategies in the field to combat TB, MDR-TB and XDR-TB, following the recommendations of the WHO and the International Union Against Tuberculosis and Lung Disease (IUATLD) [6,7].

Recently, the National Tuberculosis Reference Laboratory of Guinea-Bissau (NTRL-GB) was equipped with the GeneXpert system to be used with the Xpert MTB/RIF cartridges (Cepheid, Sunnyvale, CA, USA). This technology can detect, within two hours, directly from patients’ sputum samples, *M*. *tuberculosis* complex DNA and rifampicin resistance-associated mutations, as marker for MDR-TB, through the amplification of the entire 81 bp rifampicin resistance determining region (RRDR) of the *rpoB* gene [8]. The sensitivity of the assay for *M*. *tuberculosis* detection is equivalent to that of solid culture, and it can be used at the point-of-care levels of the laboratory network [9,10]. This assay is recommend by the WHO to be used as initial diagnostic test in individuals suspected of MDR-TB or HIV-associated TB with good analytical performances [8,9]. Thus, if mutations associated with rifampicin resistance are detected by the system, in patients considered at risk of MDR-TB, an appropriate MDR-TB therapy regimen should be started while additional sputum specimens are collected for culture and drug susceptibility testing. Subsequent testing will confirm the presence of rifampicin resistance and enable testing for drug resistance to isoniazid and other first- and second-line drugs [8,11].

The aim of this cross-sectional study with patients selected through convenience sampling was to: a) evaluate the performance and usefulness of the Xpert MTB/RIF assay for the rapid direct detection of *M*. *tuberculosis* complex in patients at risk of acquiring resistance to first-line anti-tuberculosis drugs in a limited resource setting such as Guinea-Bissau; b) assess the implications of this technology for the management of TB and drug resistant TB patients in a health care system with severe limitations to manage these patients; and c) complement the scarce information about multi and poly drug-resistant *M*. *tuberculosis* complex strains in Guinea-Bissau, through species identification, determination of the resistance profile of each strain to first- and second-line drugs, and study the genotypic diversity within the sample of recovered isolates.

## Materials and Methods

### Ethics statement

This prospective study used specimens and data collected in the course of routine patient care and resistance surveillance. All patients, informed in their native dialect about this study, provided written (or fingerprint) consent for the collection of their sputum sample and further use of results, personal information and epidemiological data. In the case of children, written informed consent was given by next of kin/caregiver. The Guinea-Bissau Government Ethics Committee for Health approved the study protocol.

### Patients enrolled and study setting

Given that Guinea-Bissau had no information on the prevalence of MDR-TB, we planned a prospective cross-sectional study with the following inclusion criteria: adult individuals (aged 15 years or older), admitted to Hospital Raoul Follereau (HRF), Bissau, Guinea-Bissau, with sputum smear microscopy positive for acid-fast bacilli (AFB) after two months of first-line anti-tuberculosis treatment, or that exhibited the criteria for re-treatment, according to the Guinea-Bissau TB control guidelines and the WHO guidelines for TB treatment [12–15]. The patients were included in this study through convenience sampling during the period from May 2012 to December 2012.

**Fig 1:**
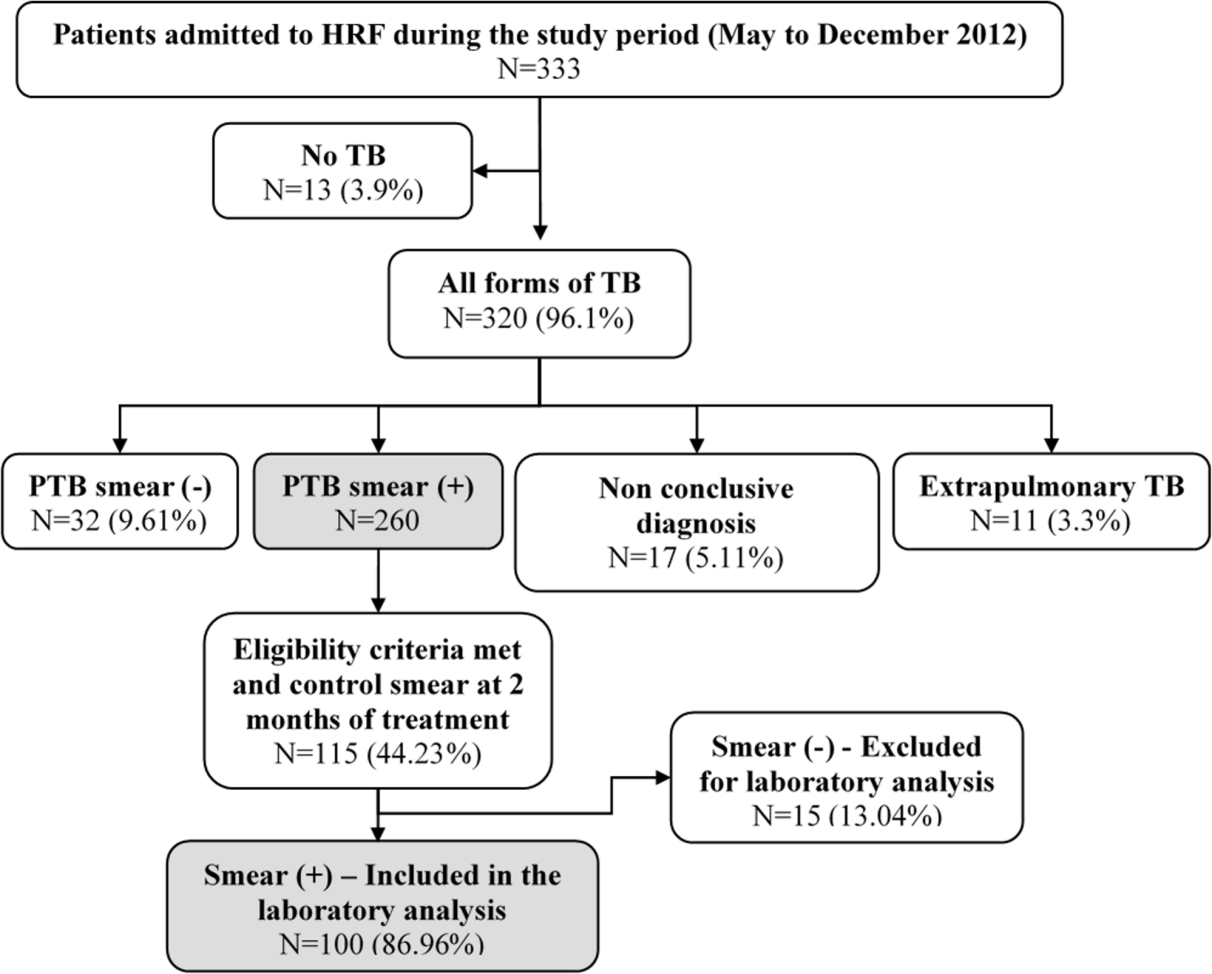
Flowchart presenting the selected study population. The diagram describes the criteria used for the selection of the patients included in the study. From the initial 333 patients admitted in the hospital with symptoms of PTB, 100 meet the inclusion criteria and were monitored in the course of the present study. HRF, Hospital Raoul Follereau; TB; Tuberculosis; PTB: Pulmonary tuberculosis; (+), positive; (-), negative.

HRF is a regional point of reference for the treatment of TB and other respiratory diseases, and the national reference center for the fight against TB. The services provided are completely free of charge for the patients in a successful public-private partnership involving the Guinea-Bissau TB national program, the national reference center in Bissau—HRF, the regional TB basic management unit and TB health centers throughout the country and a non-governmental organization—Aid, Health and Development Onlus. According to the national guidelines, the patient with TB is admitted to the HRF, after referral from regional hospitals or health centers [12,16]. During the study period, May to December 2012, a total of 333 patients with symptoms of pulmonary TB were admitted to the HRF and among them 260 were diagnosed as cases of smear positive pulmonary TB (PTB (+)); 32 as smear negative cases of pulmonary TB (PTB (-)) and 17 as inconclusive diagnosis of TB. Of the 260 patients with PTB (+), 115 were monitored in the study, since they met all the inclusion criteria mentioned above. Of these, 100 patients, all born in Guinea-Bissau, were considered as highly suspect of MDR-TB since they had their sputum smear positive at the control smear after two months of first-line treatment and were included in the subsequent complete laboratory analysis for the early detection of MDR-TB with the Xpert MTB/RIF assay. Fifteen patients were excluded from subsequent laboratory analysis because of record and smear result discrepancies between the two samples collected from the same patient and analysed in both laboratories involved (Bissau and Lisbon). After careful analysis of records and smear results from both laboratories the patients were correctly re-identified and all were confirmed as smear negative (Fig 1). The 2011 WHO implementation manuals and recommendations to technicians and clinicians for the use of the Xpert MTB/RIF assay in resource-limited settings were strictly followed [9].

### Clinical samples analysed

Between May 2012 and December 2012, a total of 200 sputum samples, two per patient, were collected from the 100 patients included in the complete laboratory analysis. Briefly, one of the sputum samples collected followed the classical laboratory analytical routine at the National Tuberculosis Reference Laboratory of Guinea-Bissau of the Instituto Nacional de Saúde Pública (INASA-GB): the smears were stained by Ziehl-Neelsen (ZN) staining technique for the detection of AFB and sample decontamination was carried by the sodium dodecyl (lauryl) sulphate method before inoculation into Lowenstein-Jensen (LJ) media that were incubated up to two months at 37°C. The other sputum sample was split into two aliquots, one for direct molecular testing and the other stored at -80°C and sent to the Mycobacteriology Laboratory of the Unidade de Microbiologia Médica, Instituto de Higiene e Medicina Tropical de Lisboa, Portugal/Universidade NOVA de Lisboa (IHMT/UNL). Sample shipment to Lisbon was made according to the International Air Transport Association (IATA) safety guidelines for infectious substances [17].

### Direct molecular detection of *Mycobacterium tuberculosis* complex

In October 2012, one GeneXpert instrument equipped with four modules was installed for the first time in Guinea-Bissau, donated to the Laboratório Nacional de Saúde Pública (INASA) of Guinea-Bissau by the Institut Pasteur of Paris, France, under the OPEC Fund for International Development grant, for the purpose of early diagnosis of MDR-TB. Upon request of INASA, a team of IHMT/UNL went to Bissau to provide additional support on software installation, implementation and guidance to use the equipment for the first time with clinical samples of patients considered at high risk for MDR-TB, included in this research project. This mission was integrated in the “Projecto de Formação em Diagnóstico da Tuberculose (ForDILAB-TB)”, supported by grants from Fundação Calouste Gulbenkian (FCG, Portugal) and the Community of the Portuguese Speaking Countries (CPLP). With the operational implementation of this system at INASA, the Xpert MTB/RIF assay was then performed directly on the previously described clinical samples collected from the patients enrolled in this study, according to the manufacturer’s instructions [8]. All RRDR mutations detected were immediately communicated to the HRF physicians, who by their turn summoned the patients for a new clinical appointment. For further genotypic studies total DNA was extracted from RRDR mutation positive sputum samples with the QIAamp DNA Mini Kit (QIAGEN, GmbH, Hilden, Germany). The INNO-LiPA Rif. TB line probe assay (Fujirebio Europe, Ghent, Belgium) was used in parallel with the Xpert MTB/RIF assay since it also allows the fast direct detection of *M*. *tuberculosis* complex and the simultaneous detection of mutations in the RRDR of the *rpoB* gene. This assay was performed as previously described during its successful application to the early detection and control MDR-TB in Lisbon [18,19].

### Sample treatment and culture isolation

As stated previously, the sputum sample aliquots were sent to Lisbon. Upon arrival, the samples were digested, decontaminated and concentrated by the standard sodium hydroxide-N-acetyl-L-cysteine (NaOH-NaLC) method [20]. Aliquots were collected for inoculation on MGIT tubes used within the BACTEC MGIT 960 (Becton-Dickinson Diagnostic Instrument Systems, Sparks, MD, USA) according to the manufacturer’s instructions [21], and the remainder of the sediment was frozen at -70°C.

When a MGIT tube was considered positive by the instrument it was further incubated at 37°C for one more day as per the manufacturer instructions. A ZN staining and inoculation in blood agar plates were done for each positive tube to confirm the presence of AFB and rule out the existence of any contamination.

### Identification of *M*. *tuberculosis* complex strains from cultures

After culture growth, the AccuProbe *M*. *tuberculosis* complex culture identification test (Gen-Probe Inc., San Diego, California) was performed according to the manufacturer's instructions and was used as the “gold standard” for the identification of *M*. *tuberculosis* complex species in the primary isolation. Because these samples were obtained from a country that has the highest prevalence of *Mycobacterium africanum* recorded in the African continent [22] and the AccuProbe *M*. *tuberculosis* complex assay is not able to discriminate the species within the *M*. *tuberculosis* complex, the Genotype MTBC assay (Hain Lifescience, Nehren, Germany) was applied for the differentiation within the *M*. *tuberculosis* complex from cultured material as recommended by the manufacturer.

### Drug susceptibility testing (DST)

First- and second-line drug susceptibility testing was performed using the proportion method in the BACTEC MGIT 960 system, according to the manufacturer's instructions [21]. The lyophilized drugs (BACTEC MGIT 960 SIRE and PZA kits; SIRE: streptomycin, isoniazid, rifampicin and ethambutol; PZA: pyrazinamide) were purchased from Becton Dickinson. Stock solutions were prepared according to the manufacturer’s instructions [21]. For the second line drug susceptibility testing the BACTEC 960 system was coupled to the Epicenter V5.80A software equipped with the TB eXIST module (Becton Dickinson Diagnostic Systems). The second line drugs used in this study and their critical concentrations were: amikacin (AMK), 1.0 μg/mL; capreomycin (CAP), 2.5 μg/mL; ofloxacin (OFX), 1.0 μg/mL; rifabutin (RBT), 0.1 μg/mL; ethionamide (ETH), 5.0 μg/mL; and paraaminosalicylic acid (PAS), 4.0 μg/mL. The stock solutions were stored at—20°C and the working solutions freshly prepared on the day of the experiment.

### Molecular typing by MIRU-VNTR and spoligotyping analysis

For the rifampicin resistant strains isolated from cultures, MIRU-VNTR genotyping was performed by multiplex PCR amplification of the 24-*loci* MIRU-VNTR, as previously described [23], in order to describe if they occurred from a recent transmission of one strain or clone or if they corresponded to genotypically unrelated strains and most probably resulted from inappropriate treatment or due to reactivation. Amplicon size determination was carried out by capillary electrophoresis on an ABI 3130 Genetic Analyzer using a custom ROX-labelled molecular weight marker, MapMarker 1200 (Bioventures), with 25 bands sized between 100–1200 bp. Amplicon calibration was previously performed by a triplicate run of the MIRU-VNTR Allelic Ladder from the MIRU-VNTR Calibration Kit (Genoscreen, Lille, France) with a custom MapMarker1200. Spoligotyping was performed as described previously [24].

A dendrogram was constructed in the public MIRU-VNTR*plus* database using the Dsw and categorical measures of genetic distance for MIRU-VNTR *loci* and spoligotyping, respectively, with equal weighting [25]. The Unweighted Pair Group Method with Arithmetic Averages (UPGMA) clustering method was used for tree construction [26,27]. Similar MIRU-VNTR and spoligotyping patterns were searched within the MIRU-VNTR*plus* and SITVIT WEB international databases [26,28].

### Data analysis

Data included in this study were: patient age, gender, nationality, TB history, HIV status, sample Xpert MTB/RIF results, INNO-LiPA Rif. TB data and first- and second-line drug susceptibility testing. Data were entered into Microsoft Office Access (Microsoft Corporation, Redmond, WA, USA) and analysed using STATA statistical software (Release 11.0, Stata Corporation, and College Station, TX, USA). Since this is a descriptive study, it was not necessary to calculate the sample size.

## Results

Of the 333 patients with symptoms of PTB admitted to the Hospital Raoul Follereau from May 2012 to December 2012, thirteen were diagnosed with diseases other than TB. Three hundred and twenty (96.1%) were diagnosed with active TB according to WHO Guidelines [14,15]. Of these, 260 (78.08%) were diagnosed as smear-positive-pulmonary TB (PTB (+)). Amongst the patients with PTB (+), 115 (44.23%) meet the inclusion criteria and were monitored in the present study. After two months of treatment, a control smear was carried out and 100 of the monitored patients were included in the complete laboratory analysis since they maintained their sputum smear-positive. Fifteen patients were excluded due to record and/or smear discrepancies between the samples analysed in both laboratories. After data revision they were confirmed as smear-negative TB (PTB (-)) and as per the approved study protocol they were excluded. The characteristics of the 100 patients enrolled in the study were as follows: (i) 74 (mean age, 37.2 years) males and 26 (mean age, 36.2 years) females; (ii) 17 HIV positive and 73 HIV negative; 4 refused to test; and 6 were not tested. Of the 17 HIV positive patients, 13 (76%) were infected with HIV-1, one (6%) was infected with HIV-2, and 3 (18%) were infected with both HIV-1 and -2. A flow diagram of the patients included in the study is presented in Fig 1.

The presence of *M*. *tuberculosis* complex DNA was detected in all 100 sputum samples tested by the Xpert MTB/RIF assay, implemented for the first time in Guinea-Bissau during the course of this study. In nine samples the molecular assay also detected mutations known to be related with rifampicin resistance (Table 1). Since the Xpert MTB/RIF assay only detects the presence of mutations, these were further characterized with the INNO-LiPA Rif. TB assay directly applied to the same samples. All strains harboured the same mutation in the *rpoB* gene, namely, the S531L mutation.

**Table 1:** Results obtained by the Xpert MTB/RIF assay.

	Xpert MTB/RIF	
	Detected	Not Detected
*M*. *tuberculosis* complex	100	0
*rpoB* RRDR mutations	9	91

Legend: MTB, *M*. *tuberculosis*; RIF, rifampicin; RRDR, rifampicin resistance determining region.

Of the 100 sputum samples analysed, 20 originated positive cultures which were identified as *M*. *tuberculosis* complex at the National Tuberculosis Reference Laboratory of Guinea-Bissau and at the Mycobacteriology Laboratory of the IHMT/UNL, Portugal. The remaining 80 samples showed no growth, most probably due to the fact that the 100 patients elected for the detailed laboratory analysis (Fig 1) were under treatment with first-line anti-tuberculosis drugs for at least two months, which rendered the TB bacilli non-viable. Of the 80 sputum samples that rendered negative cultures one had been previously identified as carrying a *rpoB* mutation for rifampicin resistance by the Xpert MTB/RIF assay and the remaining 79 were identified as *rpoB* wild-type by the Xpert MTB/RIF assay and therefore considered susceptible to rifampicin.

Eight of the nine strains that were reported by the Xpert MTB/RIF and the INNO-LiPA Rif. TB systems as carrying *rpoB* mutations associated with rifampicin resistance were isolated in culture evidencing the lack of effectiveness of the first-line anti-tuberculosis treatment. The additional sample reported to carry a *rpoB* mutation, failed to grow in any culture isolation procedure used (LJ or MGIT media). All the eight culture positive isolates carrying a *rpoB* mutation were identified as *M*. *tuberculosis* complex by the AccuProbe assay and as *M*. *tuberculosis* by the Genotype MTBC line probe assay. None of these eight strains was identified as *M*. *africanum* in spite of the reported high prevalence of this species in Guinea-Bissau [22]. However, among the remaining *M*. *tuberculosis* complex strains with positive isolation in culture, two were identified as *M*. *africanum*.

Rifampicin resistance was confirmed in the eight isolates, for which *rpoB* mutations were detected, by DST in the BACTEC MGIT 960. Additionally, resistance to other first- and second-line drugs was also detected (Table 2). The eight rifampicin resistant isolates were simultaneously resistant to isoniazid therefore fulfilling the criteria to be considered MDR-TB. Additionally, four out of these eight strains were resistant to all first-line drugs. Regarding second-line drugs, none of the MDR-TB isolates presented a XDR phenotype, as these isolates were all susceptible to amikacin, capreomycin and ofloxacin. Six MDR-TB isolates were also resistant to rifabutin, ethionamide and paraaminosalicilic acid, and two were resistant only to rifabutin (Table 2). Cross resistance between rifampicin and rifabutin, and between isoniazid and ethionamide has been well described [29,30]. In this study, all isolates showed cross resistance between rifampicin and rifabutin and six were simultaneous resistant to isoniazid and ethionamide.

**Table 2:** Clinical and laboratory data of the eight MDR-TB patients with positive Xpert MTB/RIF sputum and culture.

				First line DST					Second line DST					
Culture isolate (n = 8)	Patient HIV status	TB history: New case or retreatment*	MTB Cidentification	INH	RIF	ETB	STR	PZA	AMK	CAP	OFX	RFB	ETH	PAS
**#1**	Negative	New case	*M*. *tuberculosis*	R	R	R	R	R	S	S	S	R	R	R
**#19**	Negative	New case	*M*. *tuberculosis*	R	R	R	R	R	S	S	S	R	R	R
**#37**	Negative	New case	*M*. *tuberculosis*	R	R	R	R	S	S	S	S	R	R	R
**#52**	Negative	New case	*M*. *tuberculosis*	R	R	S	R	S	S	S	S	R	R	R
**#57**	Negative	Retreatment	*M*. *tuberculosis*	R	R	S	S	S	S	S	S	R	S	S
**#64**	Negative	Retreatment	*M*. *tuberculosis*	R	R	R	S	R	S	S	S	R	S	S
**#68**	Negative	Retreatment	*M*. *tuberculosis*	R	R	R	R	R	S	S	S	R	R	R
**#90**	Negative	New case	*M*. *tuberculosis*	R	R	R	R	R	S	S	S	R	R	R

Legend: *According to WHO criteria [14,15]; R, resistant; S, susceptible; INH, isoniazid; RIF, rifampin; ETB, etambutol; STR, streptomycin; PZA, pyrazinamide; AMK, amikacin; CAP, capreomycin; OFX, ofloxacin; RFB, rifabutin; ETH, ethionamide; PAS, paraaminosalicylic acid; MTBC, *M*. *tuberculosis* complex; TB, tuberculosis; DST, drug susceptibility testing; HIV, human immunodeficiency virus.

The limited number of rifampicin susceptible isolates obtained in this study invalidates the analysis of the accuracy of the Xpert MTB/RIF performance. Noteworthy, all the strains detected as carrying a *rpoB* mutation correlated with rifampicin resistance were confirmed as resistant by the conventional phenotypical DST. Surprisingly, all of the poly-drug and multi-drug resistant patients detected in this study were HIV negative (Table 2).

**Fig 2:**
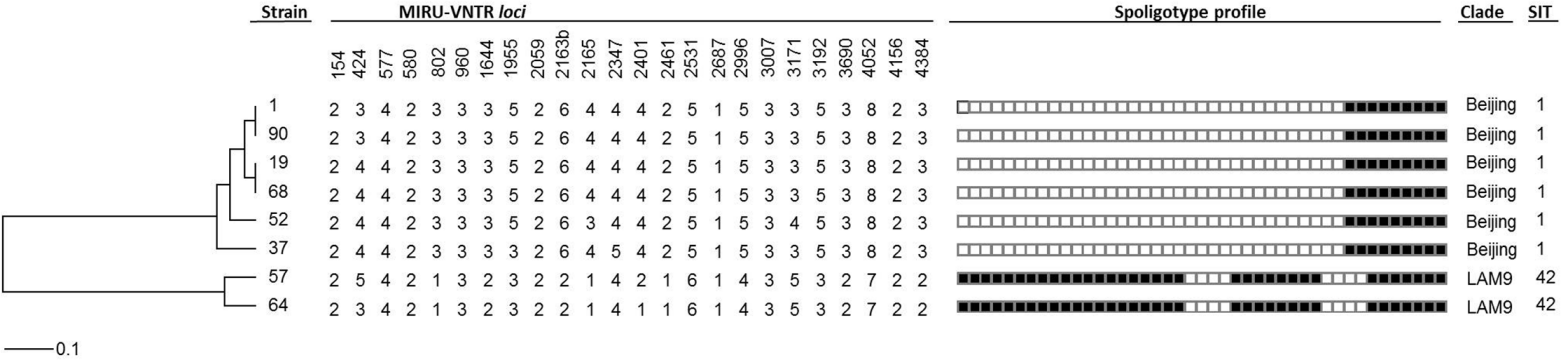
Dendogram based on the 24-*loci* MIRU-VNTR and spoligotyping profiles of the eight MDR *M*. *tuberculosis* isolates, with positive results with the Xpert MTB/RIF assay in Guinea-Bissau. The eight strains can be divided in two clades, Beijing and LAM9. Within the Beijing clade, two genetic clusters were detected; one comprised by strains #1 and #90 and the second one by strains #19 and #68. Distance scale is indicated at the bottom (see Methods for further details). SIT, shared international type (from SITVIT WEB database).

The genetic diversity of the eight MDR strains was analysed through spoligotyping and 24-*loci* MIRU-VNTR. Spoligotyping showed that the MDR strains analysed belonged to one of two genetic clades: LAM9 (2/8) and Beijing (6/8) (Fig 2). Furthermore, the MIRU-VNTR genotypic analysis allowed intra-clade discrimination by revealing distinct MIRU-VNTR profiles (Fig 2). Two clusters were detected among the Beijing clade, each comprising two isolates, which indicates recent transmission events. Moreover, three out of four of the clustered isolates were new cases, and thus primary MDR-TB cases. Nevertheless, the use of the 12-*loci* set enabled the detection of two genetic clusters comprised by the Beijing and LAM9 clades isolates, respectively (data not shown). This points to a recent common origin at each clade followed by subsequent genetic divergence that is detectable through the use of the 15-*loci* or 24-*loci* sets only. These results suggest ongoing transmission of MDR-TB strains from different origins in Guinea-Bissau and the presence of a complex and divergent transmission chain involving Beijing and LAM strains. Further studies, specifically focused on MDR-TB strains, are necessary to provide additional details on the population structure of MDR-TB strains in Guinea-Bissau.

## Discussion and Conclusions

Guinea-Bissau has been experiencing social and political instability since the 1999 civil war, ranking this West African country with one of the lowest-incomes per capita in Africa, which explains the very low health indicators, such as a high incidence rate of TB (242/100, 000 in 2013) [12]. Case detection rate for all forms of TB was only 48% (after reaching more than 80% in 1990’s), and a treatment success rate of 60% for smear-positive TB and 55% for other forms [12, 16]. As per their National Guidelines, TB patients in poor clinical conditions are admitted to HRF, after referral from regional hospitals or health centers, which receives the support of the National Tuberculosis Reference Laboratory for smear detection and occasionally isolation and identification of *M*. *tuberculosis* [12, 13, 16]. Studying a small sample of selected patients of the HRF with complete clinical records and positive sputum smears after two months of therapy, we have been able to detect 9% of MDR-TB patients and to demonstrate the feasibility and safety of implementing the Xpert MTB/RIF system in a resource-limited setting such as Guinea-Bissau with a TB control program with severe operational limitations. The early detection and monitoring of TB and MDR-TB, by molecular based techniques such as the one implemented in this study, especially when the TB laboratory cannot provide regular results on isolation, identification and drug susceptibility, was of utmost importance for the clinical and programmatic management of the patients.

Noteworthy, patient eligibility criterion in this study was smear positivity after two months of treatment as an indication of treatment failure. Eighty out of the 100 sputum samples tested rendered negative cultures. Recently, Olaru et al [31] have demonstrated that a considerable proportion of patients with culture conversion after two months of therapy continued to have detectable acid-fast bacilli on sputum smears, supporting our suggestion that the most probable cause for the smear/Xpert positive with culture negative results obtained is the non-viability of the bacilli detected. The clinical and radiographic characteristics of these patients after two months of treatment pointed towards a mild or low improvement as consequence of being initially diagnosed at very advanced stages of cavitary disease associated with difficulties in accessing and following the drug observed therapy (DOT) scheme. We also produced new data on *M*. *tuberculosis* drug-resistance resistance in this country, where field data is very scarce, and where drug susceptibility testing on first- and second-line drugs is not yet implemented.

The lack of TB conventional bacteriological laboratory services in many resource-limited settings, as is the case of Guinea-Bissau, forces many of the health systems of these countries to depend uniquely on the acid-fast smear detection as the only laboratory support available for diagnosis. Nothing can be inferred from the AFB smear about the drug susceptibility of the infecting strain, with detection and control of MDR-TB cases being based solely upon the lack of positive response from the patient to the therapy after more than two months of treatment [14,15]. After this period, in smear-positive suspected cases of MDR-TB, the patients are notified to go daily to HRF for follow up consultation. That has been the only procedure available in Guinea-Bissau to detect and monitor MDR-TB patients, clearly demonstrated in our study to be a poor indicator without concomitant laboratory indication on culture viability and/or drug susceptibility. In this study 79% of the smear-positive patients monitored had non-viable bacilli and no molecular indication of mutations for resistance to rifampicin, most probably evolving favourably after the two months of first-line treatment and not requiring changes in the implemented therapeutics, thus demonstrating the need for revision of the programmatic guidelines in use [13].

Molecular methods can improve the previous patient management problem and significantly reduce the time required for detection of drug resistance. When coupled with earlier initiation of effective therapy, they are highly effective in reducing the incidence of MDR-TB in many settings. That was the case in Portugal during the years 2000, when the systematic early direct detection of rifampicin resistance related mutations in smear positive patients, with the INNO-LiPA Rif TB line probe assay, significantly reduced the spread of MDR-TB and improved treatment outcomes [18,19]. However, the global use of line probe molecular assays for *M*. *tuberculosis* complex detection, and in particular in resource-limited settings, is rare largely due to the complexities of DNA extraction, amplification and detection, the need for sophisticated laboratory infrastructure and trained personnel and a relatively high cost. In addition, they were significantly less sensitive to culture for smear-negative TB cases [5,11].

The Xpert MTB/RIF assay, an automated hemi-nested real-time polymerase chain reaction assay for the simultaneous detection of *M*. *tuberculosis* complex DNA and mutations in the RRDR of the *rpoB* gene, was endorsed by WHO in December 2010, for the direct detection of MDR-TB in respiratory samples in patients with high risk of MDR-TB or HIV-associated TB. This system, providing results within two hours, with minimal hands-on time and minimal requirements for facilities, made molecular detection easier and accessible to resource-limited settings with the sponsorship of different health and cooperation agencies [8,9]. The excellent performance characteristics of the assay lead to its implementation in 98 high-burden and low/middle-income countries [32]. During the course of this study, the Xpert MTB/RIF was implemented in the laboratory routine of the National Tuberculosis Reference Laboratory in Bissau and its performance was very satisfactory throughout the pre-analytic, analytic, and post-analytic phases in the hands of technicians non-experienced with molecular diagnosis. As expected from the study sample (TB clinically diagnosed with smear positive sputum after two months of treatment) the Xpert MTB/RIF assay was able to identify *M*. *tuberculosis* complex in all samples and, of greater importance for the objectives of this work, the assay was able to detect nine specimens with *rpoB* mutations among the 100 specimens tested. Eight of these specimens were further confirmed by DST to be MDR cases, corresponding to a MDR-TB rate of 8% among the sample evaluated. In a setting without immediate access to either rapid laboratory testing, healthcare providers experienced in diagnosing TB or drug susceptibility assays, the Xpert MTB/RIF assay was a valuable tool to provide relevant information to the clinicians for the management of the TB patients. This was clearly perceived by the prompt adhesion of the HRF clinicians to the results and the immediate change and improvement of patient management. However, some difficulties were experienced in the implementation of this methodology, namely: a) the incomplete/almost null epidemiological data on the incidence of MDR-TB in Guinea-Bissau; b) a non-operational TB laboratory network at national level, unable to perform cultures and antibiotic susceptibility testing on a regular basis; c) the instability of the funding allocated to maintain the health system operational especially the anti-tuberculosis drug-delivery and DOT programs; d) the lack of a stable electrical system and technical support for calibration and maintenance of GeneXpert equipment. Three to four hours after the sputum collection, the suspect MDR-TB patients (with a *rpoB* mutation detected by the assay) were separated from the other patients in the hospital (no strict isolation facilities available) and proper MDR-TB management measures, according to the WHO guidelines, were immediately implemented [15].

Because confirmation of pulmonary TB relies almost solely on direct AFB-smear microscopy in Guinea-Bissau, the use of the Xpert MTB/RIF assay may increase TB case detection when applied also to smear-negative samples but a significant advantage came with the direct detection with a high level of confidence of MDR-TB in a setting where drug susceptibility tests are not available. This information was of utmost importance to improve patient management and to stop transmission of MDR-TB, being the scientific evidence upon which the health authorities decided to introduce, for the first time, the second-line drug regimen in Guinea-Bissau. Of major significance is the fact that five out of the eight confirmed MDR-TB cases diagnosed in this study had no prior record of TB or TB treatment at the time of diagnosis and were considered new TB cases corresponding to a roughly estimated 62.5% rate of primary resistance in this study. Three of these strains, belonging to the Beijing clade, were found to be clustered, which is consistent with recent transmission. On the other hand, the existence of two unclustered primary MDR-TB strains might be explained by: i) primary MDR-TB cases originated from imported MDR strains from neighbouring high-burden TB countries, and not locally transmitted, as previously reported by others [33]; ii) primary MDR-TB cases due to reactivation of an previous MDR infection; or iii) the existence of multiple MDR-TB transmission chains that can only be captured by increasing the molecular surveillance and detection of MDR-TB cases, the sample size and the study period. This latter hypothesis is likely to be the most probable explanation for these results as these strains can be clustered using the MIRU-VNTR 12 *loci* set, denoting a recent common origin. Further studies, specifically focused on MDR-TB strains, are necessary to provide additional details on the population structure of MDR-TB strains in Guinea-Bissau, further strengthening the need for the maintenance of a routine surveillance of all TB patients by molecular methods.

In conclusion, MDR-TB is an important problem for the management of TB patients in Guinea-Bissau, a low-resource and high burden TB country, requiring urgent measures to sustain the routine direct detection of the disease. Our study demonstrated that the Xpert MTB/RIF assay improved the sensitivity and time-to-diagnosis over culture based laboratory diagnosis of TB patients in Guinea-Bissau and also provided valuable information to the clinicians on the management of TB patients under treatment, allowing an efficient separation of MDR and non-MDR-TB patients. The application of the Xpert MTB/RIF assay at point-of-care and the accessibility to results within two hours after sample collection, will allow that patients suspected as having MDR-TB such as smear-positive TB patients after two months of treatment (9% of prevalence detected in this study) can be readily diagnosed at their follow-up appointment at the hospital. This will certainly reduce the time necessary to prescribe a correct treatment and implement isolation measures in order to reduce the transmission of MDR *M*. *tuberculosis* strains into the community. However, this molecular system can only be effective if its financial and technical sustainability and real translation onto improvement of patient’s outcomes can be assured in the future [34]. Furthermore, the implementation of molecular detection techniques do not preclude in any way the need for a routine quality assured TB laboratory providing phenotypic based full reports on strains isolated from TB and MDR-TB patients to guarantee the most adequate treatment to these patients [8, 18, 32].
